# Proteomic and metabolomic profiling of methicillin resistant versus methicillin sensitive *Staphylococcus aureus* using a simultaneous extraction protocol

**DOI:** 10.3389/fmicb.2024.1402796

**Published:** 2024-06-26

**Authors:** Syrine Boucherabine, Alexander Giddey, Rania Nassar, Hamza M. Al-Hroub, Lobna Mohamed, Mohammad Harb, Nelson Cruz Soares, Abiola Senok

**Affiliations:** ^1^College of Medicine, Mohammed Bin Rashid University of Medicine and Health Sciences, Dubai, United Arab Emirates; ^2^Center for Applied and Translational Genomics, Mohammed Bin Rashid University of Medicine and Health Sciences, Dubai, United Arab Emirates; ^3^School of Dentistry, Cardiff University, Cardiff, United Kingdom; ^4^Research Institute of Medical and Health Sciences, University of Sharjah, Sharjah, United Arab Emirates; ^5^Department of Medicinal Chemistry, College of Pharmacy, University of Sharjah, Sharjah, United Arab Emirates; ^6^Laboratory of Proteomics, Department of Human Genetics, National Institute of Health Doutor Ricardo Jorge (INSA), Lisbon, Portugal; ^7^Centre for Toxicogenomics and Human Health (ToxOmics), NOVA School/Faculdade de Lisboa, Lisbon, Portugal

**Keywords:** proteomics, metabolomics, mass spectrometry, *Staphylococcus aureus*, gram positive bacteria

## Abstract

**Background:**

Understanding the biology of methicillin resistant *Staphylococcus aureus* (MRSA) is crucial to unlocking insights for new targets in our fight against this antimicrobial resistant priority pathogen. Although proteomics and metabolomic profiling offer the potential to elucidating such biological markers, reports of methodological approaches for carrying this out in *S. aureus* isolates remain limited. We describe the use of a dual-functionality methanol extraction method for the concurrent extraction of protein and metabolites from *S. aureus* and report on the comparative analysis of the proteomic and metabolomic profiles of MRSA versus methicillin sensitive *S. aureus* (MSSA).

**Methods:**

Bacterial reference strains MRSA ATCC43300 and MSSA ATCC25923 were used. The conventional urea methodology was used for protein extraction and a methanol based method was used for concurrent proteins and metabolites extraction. Proteomic and metabolomic profiling was carried out using TimsTOF mass spectrometry. Data processing was carried out using the MaxQuant version 2.1.4.0.

**Results:**

This study represents the first report on the utilization of the methanol extraction method for concurrent protein and metabolite extraction in Gram positive bacteria. Our findings demonstrate good performance of the method for the dual extraction of proteins and metabolites from *S. aureus* with demonstration of reproducibility. Comparison of MRSA and MSSA strains revealed 407 proteins with significantly different expression levels. Enrichment analysis of those proteins revealed distinct pathways involved in fatty acid degradation, metabolism and beta-lactam resistance. Penicillin-binding protein PBP2a, the key determinant of MRSA resistance, exhibited distinct expression patterns in MRSA isolates. Metabolomic analysis identified 146 metabolites with only one exclusive to the MRSA. The enriched pathways identified were related to arginine metabolism and biosynthesis.

**Conclusion:**

Our findings demonstrate the effectiveness of the methanol-based dual-extraction method, providing simultaneous insights into the proteomic and metabolomic landscapes of *S. aureus* strains. These findings demonstrate the utility of proteomic and metabolomic profiling for elucidating the biological basis of antimicrobial resistance.

## Introduction

Proteomic and metabolomic profiling is a promising research area which has been applied in studies investigating the pathological mechanisms underpinning serious conditions including viral infections such as COVID-19 (Bi et al., 2022; Costanzo et al., 2022). The application of proteomics and metabolomics approaches for the study of microbial agents has the potential to elucidate a comprehensive profile of their metabolic functions and provide novel insights into their biological characteristics. In addition, novel markers which could be used as targets for therapeutics and diagnostics can also be identified. Thus, the utilization of this approach in investigating antimicrobial resistant (AMR) pathogens is particularly pertinent.

*Staphylococcus aureus* is a ubiquitous human pathogen and methicillin-resistant *S. aureus* (MRSA) which emerged in the early 1960s is now a global concern and listed as a priority AMR pathogen by the World Health Organization (Who.int, n.d.). The molecular epidemiology of MRSA remains dynamic as shown by data from genomic studies (Senok et al., 2019, 2020a,b), however, metabolic markers which could be correlates for resistance phenotype, drivers of transmission and pathogenicity are yet to be fully elucidated. In light of this, proteomics and metabolomics profiling of methicillin sensitive *S. aureus* (MSSA) and MRSA isolates could provide much-needed insights into microbial biology and drug resistance mechanisms. Currently reported investigations of the proteomic profiling of *S. aureus* have focused on the comparison of planktonic cells and biofilms, resistant and sensitive strains, or the response of isolates to diverse stress conditions (Resch et al., 2006; Liebeke et al., 2011; Liu et al., 2014; Atshan et al., 2015; Rahman et al., 2022; Sulaiman et al., 2022). These studies utilized urea extraction which is considered the method of choice for protein extraction in microbial proteomics. However, the urea extraction method does not enable concurrent extraction of metabolites.

The application of a methodology with dual functionality allowing for concurrent protein and metabolite extraction on samples from bacteria colonies is extremely attractive. A previous report utilizing such an approach was reported by Fortuin et al. which enabled the proteomic and metabolomic profiling of *Escherichia coli* (Fortuin et al., 2020). Currently, there is no report in the literature describing the concurrent extraction of proteins and metabolites in Gram positive bacteria. In addition, to the best of our knowledge, data on the metabolomic profiling of MSSA versus MRSA isolates is also lacking. Developing such an extraction method is promising for the advancement of proteome and metabolome studies of Gram positive bacteria. In light of this, our study was designed to investigate the efficacy of a dual extraction method for downstream metabolomic and proteomic profiling of Gram positive bacteria. Furthermore, through the utilization of MSSA and MRSA isolates for this investigation we sought to provide novel insight into the metabolomic profile of a clinically relevant pathogen.

## Methods

### Bacterial culture

Bacteria reference strains used in this study were MRSA ATCC 43300 and MSSA ATCC25923. Briefly, fresh cultures were prepared from frozen stocks on Colombia blood agar. Full loop of cells were scraped and resuspended in 300 μL of distilled water, then centrifuged for 2 min at 14600 g, water was discarded and cells were washed twice with PBS. Lastly, cell pellets were frozen in −20°C.

### Urea method for protein extraction

We utilized the conventional urea methodology for protein extraction (Sulaiman et al., 2021). Briefly, original cell pellets were resuspended in a urea-based lysis buffer (8 M Urea, 50 mM Tris–HCL) (pH 8), and protease inhibitor cocktail tablets (Roche, Mannheim Germany) as previously described (Sulaiman et al., 2021). Samples were then placed in liquid nitrogen for 10 s and sonicated for 10 min in a water bath sonicator (30 s on, 30 s off, 10 cycles at 4°C). Samples were then centrifuged at 4°C for 15 min at 14600 g, and supernatant was then transferred to a new labelled microcentrifuge tube and placed on ice. Proteins were quantified using the modified Bradford protein quantification assay as previously described and each sample was adjusted to a final total protein concentration of 20 μg (Ramagli and Rodriguez, 1985). All samples were prepared in quadruplates.

### Methanol method for dual extraction of proteins and metabolites

A methanol based extraction method was used for concurrent extraction of proteins and metabolites from the same sample (Zenati et al., 2023). The first step of this technique enables acquisition of the metabolites followed by the protein extraction.

Metabolite extraction: cell pellets were resuspended in 300 μL of cold methanol and kept at −20°C for 2 h. Samples were then flash frozen in liquid nitrogen for 10 s and sonicated in a waterbath sonicator for 10 min (30 s on, 30 s off, for 10 cycles at 4°C). Samples were centrifuged at 4°C for 15 min at 14600 g. Supernatant was transferred to a new labelled microcentrifuge tube, and dried using speed vac at 38°C and stored in −80°C pending analysis. The remaining cell pellet was used for protein extraction. For the analysis for metabolites, quadruplicates were prepared for each reference strain. Briefly, the dried metabolite samples were resuspended in 200 μL of 0.1% formic acid solution and filtered through a hydrophilic nylon syringe filter (0.45 μm pore size).

Protein extraction: the cell pellet from the metabolite extraction were resuspended in the urea containing lysis solution as used for the direct protein extraction method. The samples were centrifuged at 14600 g for 15 min at 4°C. The supernatant was moved to a new microcentrifuge tube and the remaining cellular debris pellet discarded. Proteins extracted were then quantified using the modified Bradford protein quantification assay.

### Tryptic digestion

Tryptic digestion was carried out for the protein extracted by both methodologies described above. We prepared solutions containing 20 μg of extracted protein which were incubated with 1 mM dithiothreitol (DTT) for 1 h with gentle shaking at room temperature to break intra- and inter-protein disulphide bonds. This was followed by alkylation with 50 mM iodoacetamide (IAA) for 30 min. Finally, trypsin was added at a final concentration of 1:50 and left overnight at room temperature. Digestion was stopped by adding Trifluoroacetic acid (TFA) at a final concentration of 1%. The solutions containing peptides were then desalted using C18 tips (Zenati et al., 2023) and dried down using the Speed vac at 38°C. For analysis, samples were reconstituted in a solution made of 0.1% formic acid (FA) and 2% acetonitrile (ACN).

### LC-MS/MS analysis

#### Proteomics profiling

This was carried out using the TimsTOF mass spectrometry and a Nano Elute UHPLC and CaptiveSpray ion source (Bruker, Darmstadt, Germany). For the nanoflow high-performance liquid chromatography (HPLC) analyses, 4 μL aliquots (4 μg peptides) were injected and separated along a FIFTEEN^®^ C18 column (15 cm × 75 μm, 1.9 μm) (Bruker, Darmstadt, Germany) with 140 min total run time. Solvent A was 0.1% formic acid in LC-MS grade water, and solvent B was 0.1% formic acid in ACN and the gradient was held at 5% B for 5 min, then gradually ramped to 35% B over 115 min. The column was then washed and re-equilibrated by rapidly ramping to 95% B over 5 min, holding at 95% B for 10 min, then re-equilibrating at 5% B for 5 min. The flow rate was operated at 300 nL/min, the drying gas at 3 L/min at a temperature of 150°C and the capillary voltage was +1.6 kV. The scan range was set to 150–2,200 m/z and the instrument was operated in auto-MS/MS mode with CID fragmentation. The collision energy was set to vary by precursor m/z between 23 eV and 65 eV. A fixed cycle time of 3 s was used with a minimum relative intensity threshold of 500 counts per thousand and a target intensity of 10,000 counts and analyte charge was required to be 2 ≤ *x* ≤ 5. Precursor scans were performed at 2 Hz and active exclusion was triggered after acquisition of one spectrum and released after 0.4 min. For each extraction method, the samples were run in quadruplicates.

#### Metabolomics profiling

For metabolomics, 10 μL was injected twice for each sample and eluted using a 30 min gradient as follows: 1% ACN was held for 2 min, ramping to 99% ACN over 15 mintes, held at 99% ACN for 3 min before re-equilibrating to 1% ACN for 10 min. Flow rates were 250 μL/min for elution and 350 μL/min for re-equilibration. The MS analysis was performed using a TimsTOF (Bruker, Darmstadt, Germany) with Apollo II electrospray ionization (ESI) source. The drying gas was set to flow at 10 L/min and the drying temperature to 220°C and the nebulizer pressure to 2.2 bar. The capillary voltage was 4,500 V and the end plate offset 500 V. The peak intensities were log2 transformed and only the metabolites present in at least 70% of the samples of at least one group were retained for statistical testing. The remaining missing values were imputed by half of the minimum value observed throughout the dataset.

#### Data processing and statistical analysis

Raw “.d” files were processed with MaxQuant version 2.1.4.0 for feature extraction and peptide and protein assignments made using the Andromeda search engine using the Uniprot proteome for *S. aureus* (UP000054329, 2,937 entries, 14 September 2023). For the MS/MS database search, the default parameter settings were applied, with carbamidomethylation of cysteine residues set as fixed modification and acetylation of protein N-termini and methionine oxidation assigned as variable modifications. Peptide spectral matches (PSMs) were filtered with a 1% false discovery rate (FDR) using a target-decoy approach and a 20 ppm precursor mass tolerance. The default trypsin/P enzymatic cleavage rule was used for *in silico* digestion and the MaxLFQ algorithm was used for label-free quantitation (LFQ) with a minimum of two shared peptides required.

**Figure 1:**
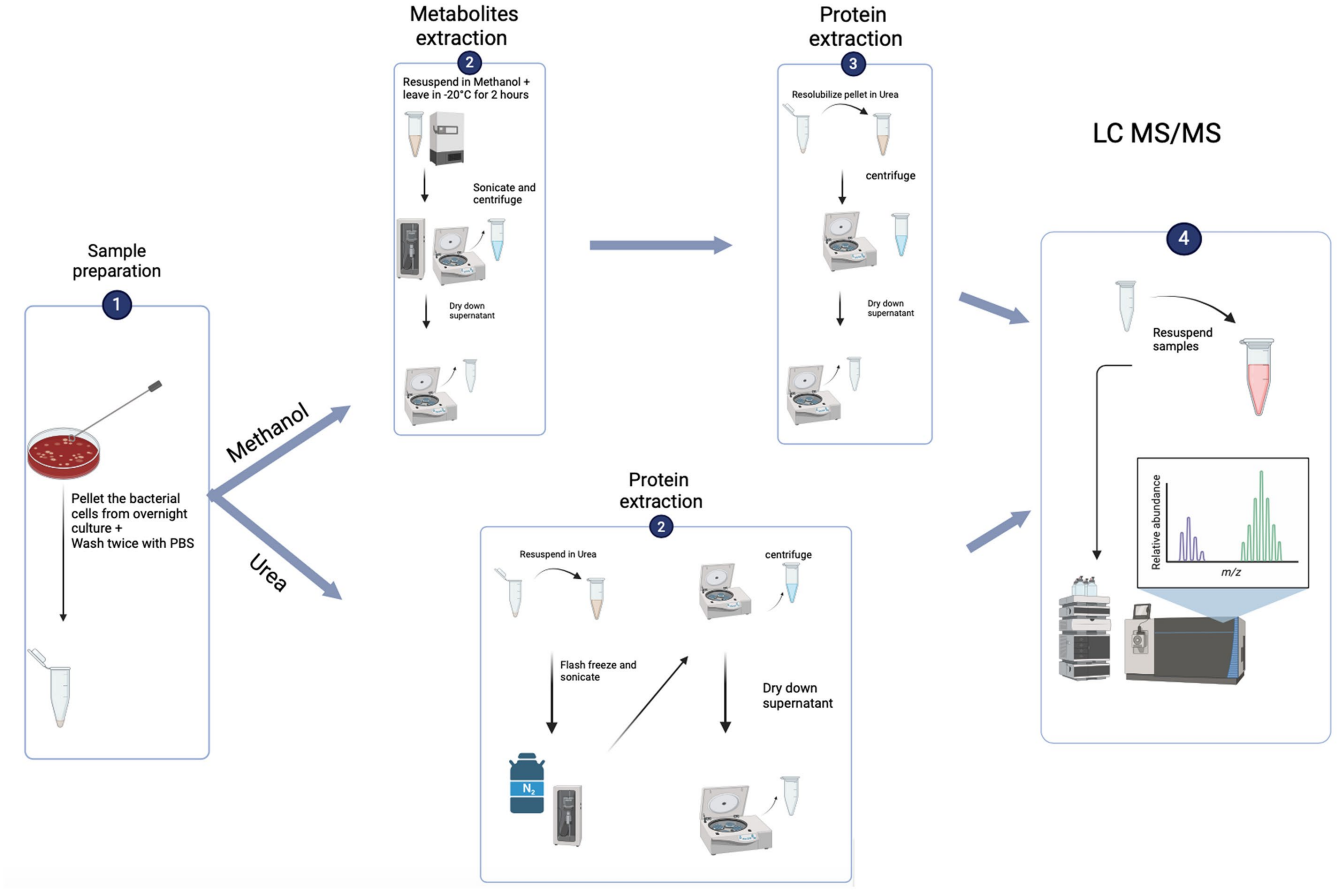
Flowchart of the experiment set up (created using biorender.com).

Protein hits to the target database were retained, while those matching to potential contaminants were removed prior to analysis. LFQ intensities were log_2_-transformed and only those protein groups present in at least 70% of the samples of at least one group were retained for statistical testing. The remaining missing values were imputed by half of the minimum value observed throughout the dataset. Significance was determined using Student’s *t*-test with *p* < 0.05 after correcting for multiple comparisons by the Benjamini-Hochberg FDR method. Analysis was performed in R using the packages “prcomp” for principal components analysis, “pheatmap2” for hierarchical clustering and heatmap visualisations, and “ggplot2” and “ggpubr” for protein abundance profiles. Gene set enrichment analysis (GSEA) was performed using “clusterProfiler” and significance was determined by a post-Benjamini-Hochberg adjusted *p* < 0.05 (See Figure 1).

## Results

**Figure 2:**
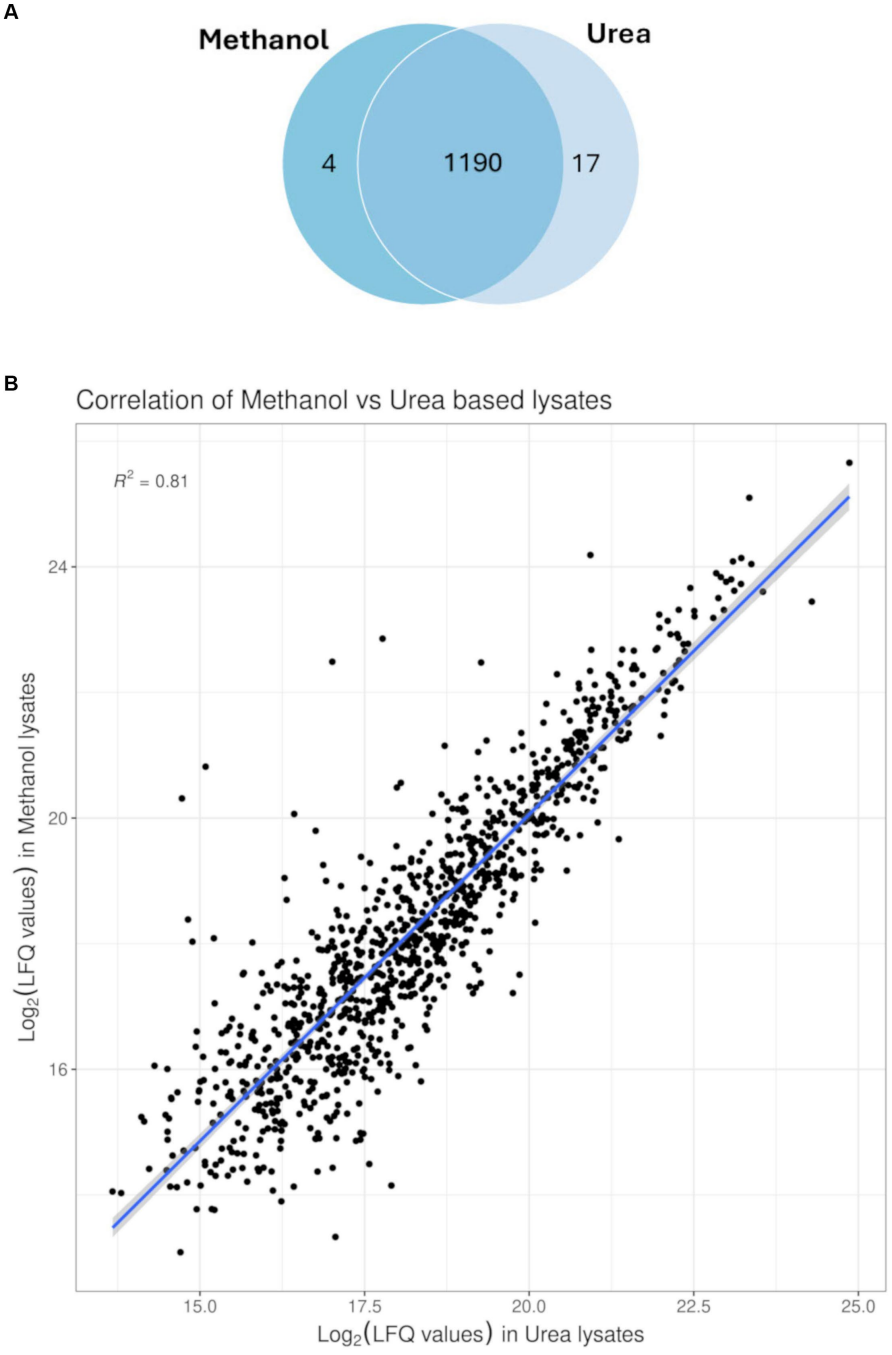
Profiling of proteins identified. **(A)** Venn diagram of the overlap as well as unique proteins identified from MRSA ATCC43300 in either of the extraction methods, with 1,190 protein found in both extraction methods and only 4 proteins exclusive to the methanol extraction method compared to 17 proteins exclusive to the conventional urea extraction method. **(B)** Correlation plot between both extraction methods (Pearson correlation coefficient 0.8; *p* < 0.05).

To compare efficacy and reproducibility of protein extraction by the conventional urea method (Sulaiman et al., 2021) and the newly applied methanol method, we analysed the proteome of MRSA ATCC 43300 reference strain using an un-targeted shotgun proteomic approach. For each extraction method, the overlap of identified peptides and proteins as well as variability in intensities within sample was compared. Pearson correlation was run between technical replicates and coefficient of variance was determined for each method. Both extraction methods showed high technical reproducibility with the urea method showing an average CV value of 10.9%, and the methanol method an average CV value of 20.5%. Global proteomics analysis showed the detection of 1,211 proteins across both methods. A total of 15,217 peptides were observed with a median of 9 assigned peptides per protein, and 58% of the proteins had zero missed cleavages. Comparative analysis of identified proteins showed 1,196 proteins between both methods with only 5 unique proteins for the methanol method and 12 unique proteins for the urea method (Figure 2A). The unique proteins identified in each method is shown in Table 1. To assess the quantitative variability, we compared the coefficient of variance for the two methods. The Pearson correlation coefficient was 0.8, indicative of a strong positive correlation between both methods (*p* < 0.05), (Figure 2B).

**Table 1:** List of the unique MRSA proteins identified in the dual methanol method and the conventional urea method.

Methanol method	
	NETI family protein
	Adhesin
	Exodeoxyribonuclease 7 small subunit
	Ohr family peroxiredoxin
Urea method	
	Urease subunit gamma
	Acetyltransferase
	Regulatory protein RecX
	4-hydroxy-tetrahydrodipicolinate reductase
	Amidohydrolase
	ATP-dependent DNA helicase RecG
	Cysteine protease
	Type VII secretion system protein EssC
	3-dehydroquinate synthase
	DUF536 domain-containing protein
	CidR
	Beta-hemolysin
	Cysteine protease inhibitor (Staphostatin A)
	Na+ dependent nucleoside transporter domain-containing protein
	Lipoprotein
	Octanoyl-[GcvH]:protein N-octanoyltransferase
	HAD family hydrolase

**Figure 3:**
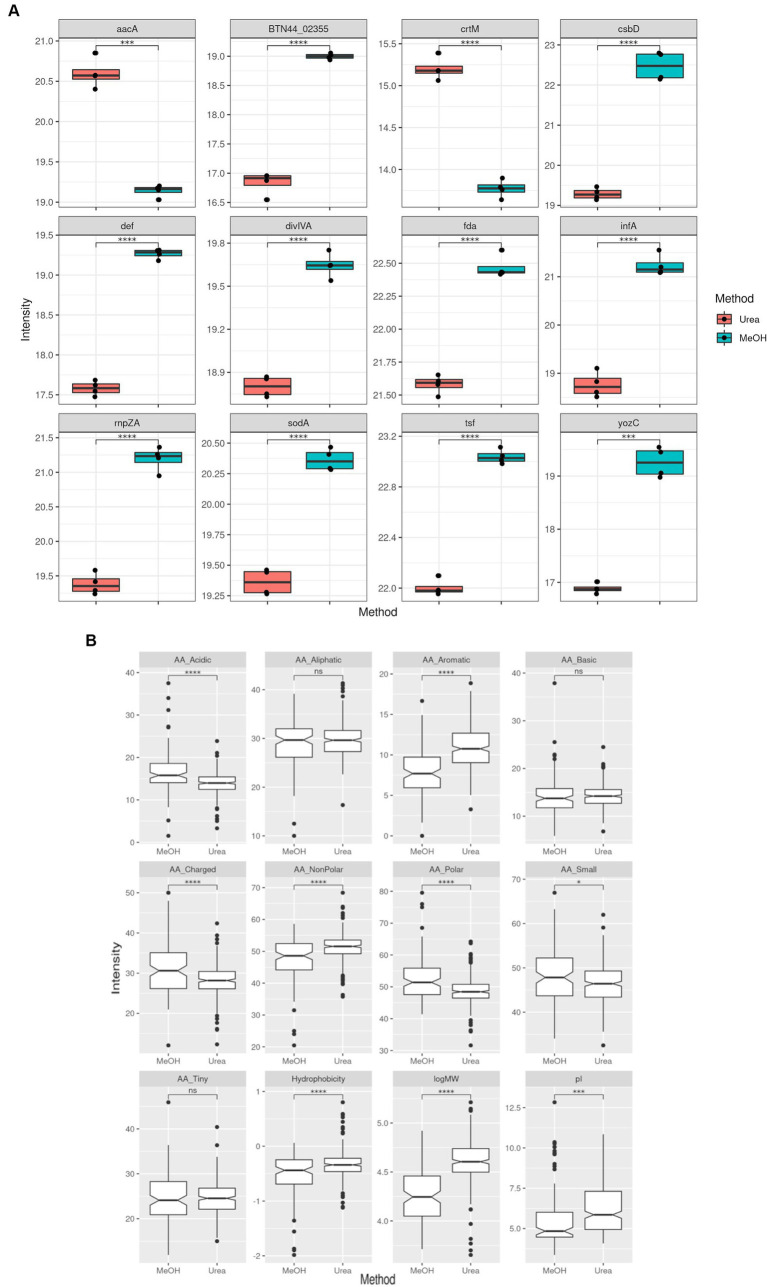
Comparison of MRSA protein label-free quantitation (LFQ) intensities obtained using both extraction methods. **(A)** Boxplots representing the top 12 proteins with differential LFQ levels. **(B)** Boxplots representing the differences between the properties of the protein with different LFQ levels.

After establishing the quantitative variability between both methods, we then evaluated the proteins that were present at different intensity levels. Comparison of the two extraction methods showed that there were 407 proteins with significant differences in abuandance depending on the extraction method (*p* < 0.05) (See Figure 3).

### Application of the methanol method for comparison of proteomic and metabolomic profiling of MRSA versus MSSA strains

**Figure 4:**
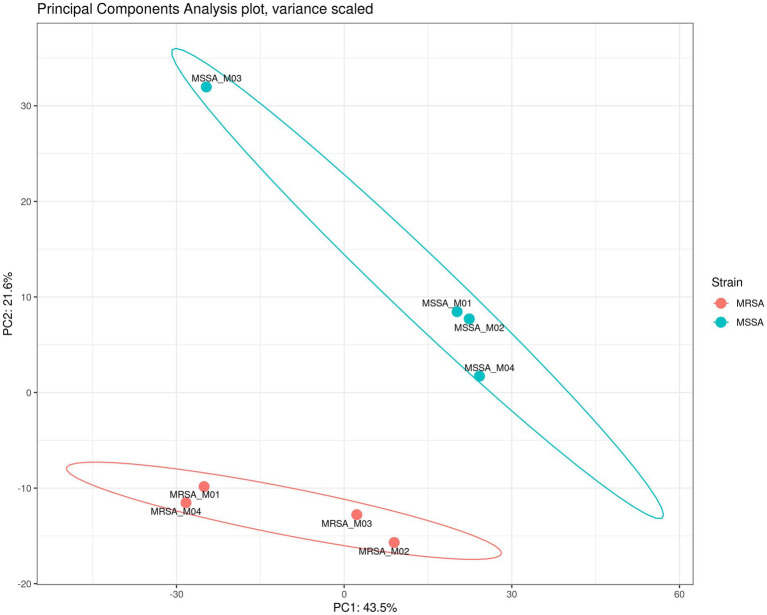
PCA of the proteomes of MSSA vs. MRSA and their respective replicates showing the clustering together of strains.

**Figure 5:**
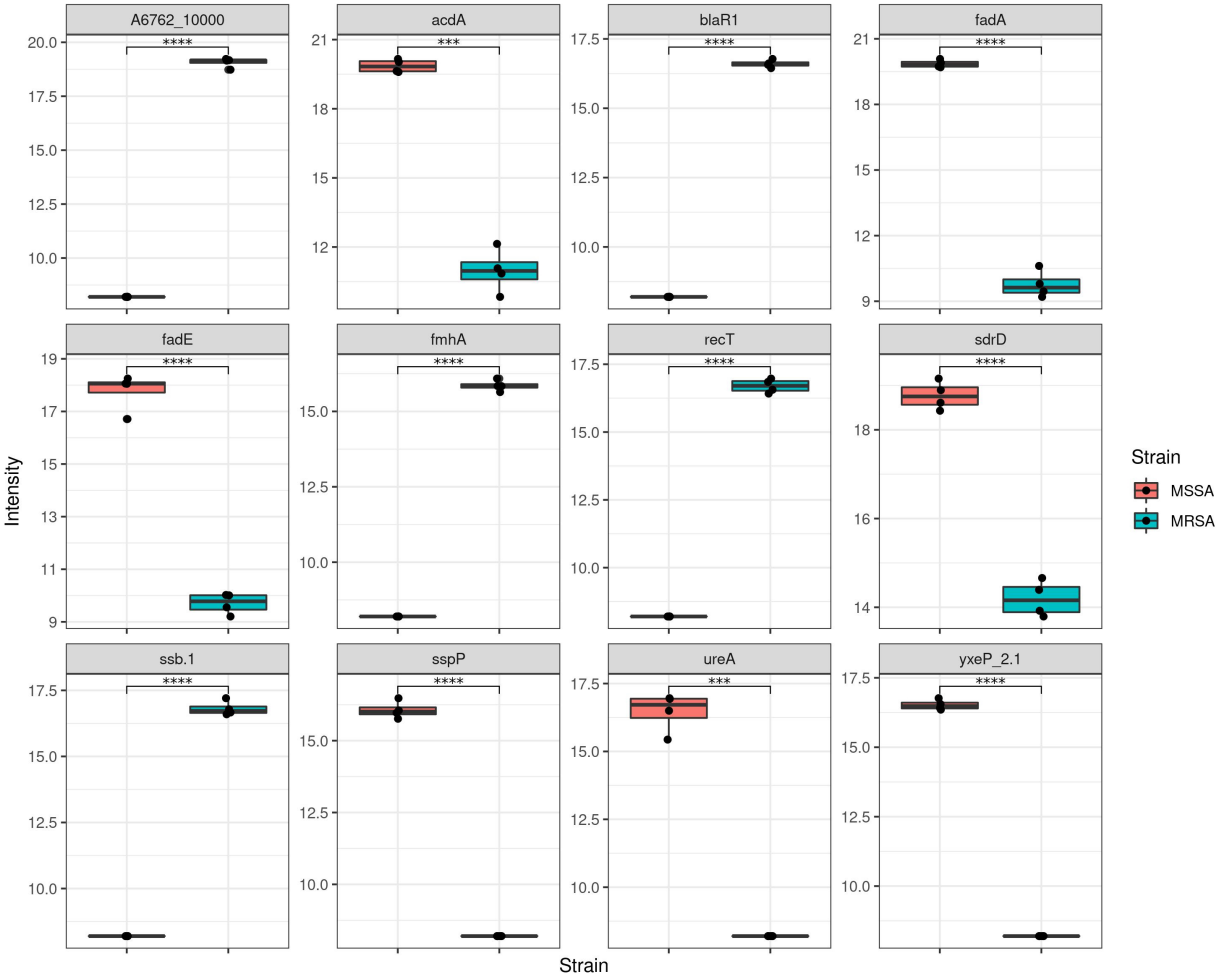
Comparison of LFQ intensities of methicillin sensitive and resistant *Staphylococcus aureus* (MSSA and MRSA).

To assess the application of the methanol method, comparative evaluation of the proteomic and metabolomic profiles of MSSA and MRSA extracted using this method was carried out. A total of 1,211 proteins were identified across both MRSA and MSSA strains with 58% having zero missed cleavages. The number of peptides and average peptide number per protein was similar in both strains. The principal component analysis (PCA) showed that all replicates from the same strain were clustered together, with distinct separation between the proteomes of both groups (Figure 4). To further validate the strength of the methanol method, comparative analysis of findings for MRSA versus MSSA strains was carried out. For the proteomic profiling, the main differences observed between both strains are shown in Figure 4. There were 77 proteins with differential LFQ levels between MRSA and MSSA strains (Figure 5). Enrichment analysis and protein–protein interaction using String-db of the 77 proteins with differential LFQ abudance identified 5 KEGG pathways linked with amino acid metabolism, fatty acid degredation and beta-lactam resistance (Table 2).

**Table 2:** Enriched KEGG pathways of the identified protein with different LFQ values.

	Observed gene count	Background gene count	Strength	False discovery rate
Metabolic pathways	42	915	0.24	0.0027
Glycine, serine and threonine metabolism	7	38	0.85	0.0066
Microbial metabolism in diverse environments	15	200	0.46	0.0100
Fatty acid degradation	4	14	1.04	0.0271
Beta-Lactam resistance	6	45	0.71	0.0389

All pathways were found to be more enriched in MSSA with the exeption of the beta-lactam resistance pathway.

### Metabolomic analysis

**Figure 6:**
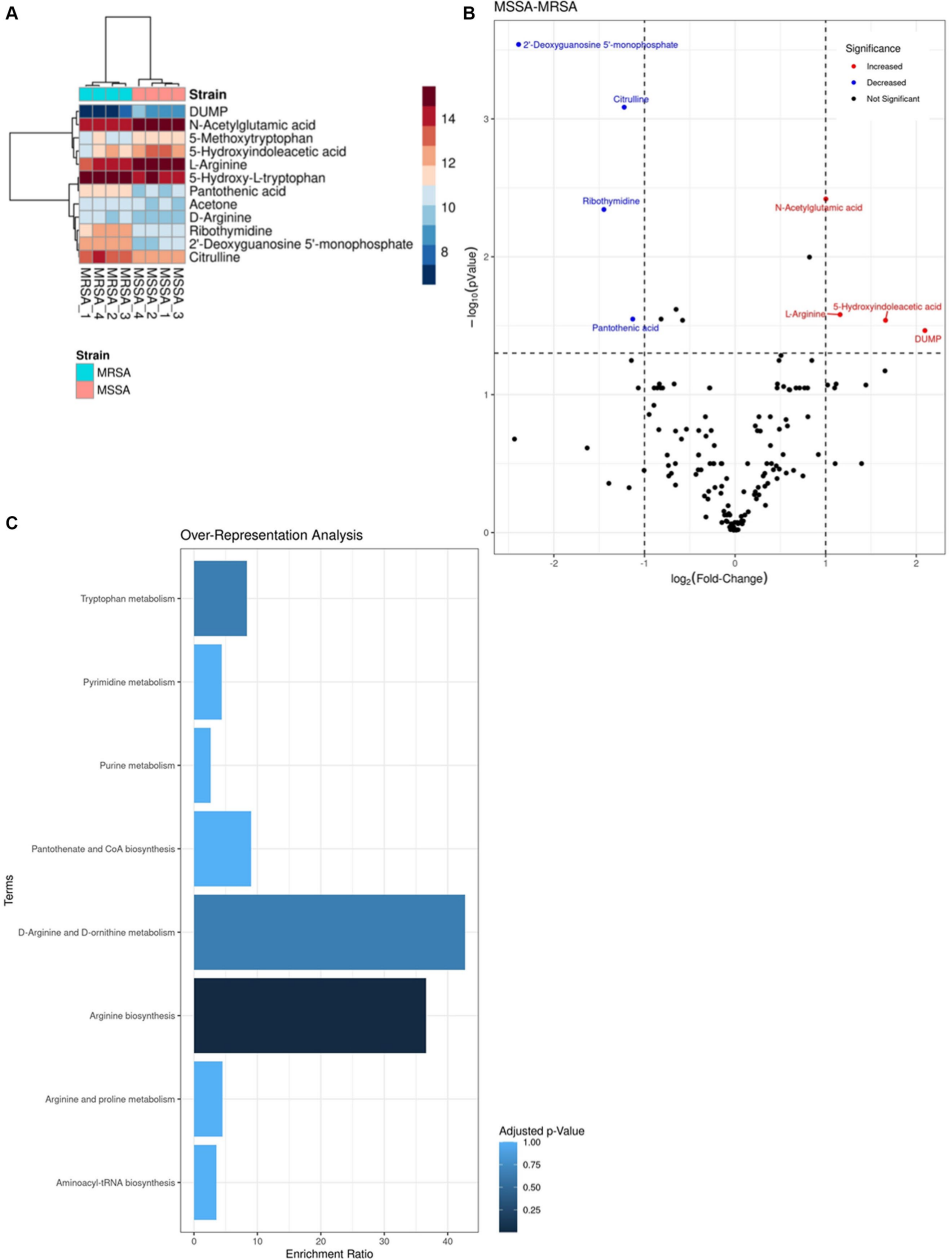
Comparison of LFQ intensities of the top metabolites identified in methicillin sensitive and resistant *Staphylococcus aureus* (MSSAA and MRSA). **(A)** Heatmap of the differences in LFQ intensities between top metabolites identified in both strains. **(B)** Volcano plot representing the LFQ values of shared metabolites between both strains, indicating a log-2 fold change in intensity. Red dots represent higher intensity in MSSA compared to MRSA, while blue dots represent lower intensity in MSSA. **(C)** Over-representation analysis of enriched pathways done on KEGG based on metabolites identified.

As the advantage of the methanol method is the dual functionality of concurrent extraction of both metabolites and proteins from the same sample, we analyzed the metabolomic profiles of the MRSA and MSSA reference strains. Data of four biological replicates of each strain were used for the analysis. A total of 146 metabolites were identified for both strains. Most of the metabolites were identified across both phenotypes with varying intensity levels, with only one metabolite being exclusive to the resistant phenotype (Figures 6A,B). The 4 metabolites with higher intensity in MSSA were L-Arginine, DUMP, Hydroxyindoleacetic acid, N-Acetylglutamic acid whilst those showing higher intensity in MRSA were Pantothenic acid, Ribothymidine, Citrulline, 2’-Deoxyguanosine 5′-monophosphate. Enrichment analysis of metabolites with dysregulated intensities was carried out using KEGG. A total of eight enriched pathways were identified (Figure 6C) with D-arginine and D-ornithine metabolism and arginine biosynthesis being the most enriched ones.

## Disscussion

*S. aureus* is an important agent of community acquired and nosocomial infections, as well as an AMR priority pathogen. Majority of studies conducted on *S. aureus* have focused on the phenotypic and molecular resistance profiles with a paucity of data on comparative proteomic and metabolomic profiling of MSSA and MRSA strains (Senok et al., 2019, 2020a,b). To bridge this gap in the literature, we investigated the use of a single-phase methanol extraction method which enabled concurrent extraction of proteins and metabolites from *S. aureus* isolates. We then validated it by utilizing mass spectrometry-based untargeted proteomics and metabolomics to compare the protein and metabolite expression profile of MSSA and MRSA.

This dual functionality approach incorporates utilization of methanol into the conventional urea extraction method to facilitate metabolite extraction (Alshehry et al., 2015; Wong et al., 2019; Zenati et al., 2023). Previously reported work using this approach was carried out using the Gram negative bacteria *E. coli* (Ramagli and Rodriguez, 1985). Gram positive bacteria are traditionally are recognized as being difficult to lyse in comparison to Gram negative bacteria which tends to raise methodological challenges in extraction protocols (Auer and Weibel, 2017). This study represents the first report on the utilization of the methanol extraction method for concurrent protein and metabolite extraction in Gram positive bacteria. Our findings demonstrate that this method performed very well for the dual extraction of proteins and metabolites from *S. aureus* with demonstration of reproducibility. Specifically for the extraction of proteins, this method also showed comparable results to the conventional urea extraction method. Whilst there were few proteins which were exclusive to each extraction method, there was no distinct pattern of high significance associated with these proteins. However, it was observed that larger proteins were recovered at a higher intensity with the urea extraction method, which suggests that it could be a more useful approach for investigations aiming for extraction of high molecular weight proteins. Additionally, although the use of organic solvents such as methanol has been reported to be more efficient for the recovery of membrane proteins and transmembrane peptides (Gowda and Raftery, 2014; Al-Salhi et al., 2022; Alsoud et al., 2022), our findings did not show any differences between the two methods in the yield of these hydrophobic proteins.

Previous work done on MRSA has demonstrated the effect of various environmental conditions and stress on its proteomic profile and how those changes allow MRSA to adapt to the harsh environment and thrive in it (Resch et al., 2006; Liebeke et al., 2011; Liu et al., 2014; Atshan et al., 2015; Rahman et al., 2022; Sulaiman et al., 2022). However, data on the comparative evaluation for the proteomic and metabolomic profile of MRSA and MSSA isolates are lacking. Indeed, our review of the literature failed to identify published work comparing MRSA and MSSA isolates using untargeted proteomic and metabolic anlaysis. In this study, of the 1,211 protein identified, 77 showed significant differences in abuandance between MRSA and MSSA. Significantly in the MRSA isolate, we identified several proteins associated with antibiotic resistance, including penicillin-binding protein PBP2a, known for its pivotal role in methicillin resistance. Additionally, proteins related to virulence factors, biofilm formation, and stress response mechanisms exhibited significant variations. Enrichment anlysis of KEGG pathways of proteins with significant changes in their LFQ revealed 5 enriched pathways including those for microbial metabolism, fatty acid degradation, beta-lactam resistance, microbial metabolism in diverse environments and amino acids metabolism.

Microbial metabolism is one of the main processes conducted by bacteria as it uses it to break down nutrients and obtain energy, to ensure its survivability and fitness. This process is inclusive of various pathways such as those pertaining to the tricarboxylic acid (TCA) cycle (Tollerson and Ibba, 2020). The regulation of these pathways is crucial to the bacteria in adaptation to outside stimuli and diverse environmental factors toward ensuring bacterial survival throughout various dynamic host and environmental changes (Tollerson and Ibba, 2020). Our findings showed that pathways associated with microbial metabolism which were significantly enriched in MSSA compared to MRSA include those responsible for fatty acid degradation as well as pathways for glycine, serine and threonine metabolism. Fatty acids along with amino acids are considered energy sources and are used by *S. aureus* as key source of carbon for ATP synthesis in the absence of glucose, making it an important survival mechanism under adverse conditions (Halsey et al., 2017). Morever, reports have also shown the tendency of serum-resistant bacteria, which tend to be antibiotic resistant, to downregulate the glycine, serine and threonine metabolism pathways (Cheng et al., 2019). Therefore, the enrichment of these pathways by MSSA might be suggestive of higher fitness in this sensitive *S. aureus* isolate. This finding is in concordance with previous reports demonstrating that sensitive strains tends to outrank resistant ones in an antibiotic free environment as well as loss of bacterial fitness with acquisition of resistance genes (Rajer and Sandegren, 2022).

The penicillin-binding protein family is a family of transpeptidase enzymes involved in cell wall synthesis in bacteria and the target of beta-lactams antibiotics (Bush and Bradford, 2016; Lade and Kim, 2023). Expression of penicillin-binding protein 2a (PBP2a), encoded by the *mecA* gene, with a lower affinity to beta-lactams constitutes the resistance mechanism associated with the MRSA phenotype (Hartman and Tomasz, 1984). Our findings demonstrate that the beta-lactam resistance pathway was more enriched in MRSA, which is in concordance with reported genomic and phenotypic studies (Senok et al., 2019, 2020a,b). Furthermore, abundance of PBP2a in MRSA isolates is expected and could be used as performance indicator of a new extraction method. The findings from our proteomics analysis demonstrated this expected high abundance of PBP2a in the MRSA isolate.

The comparative metabolomic analysis identified the arginine metabolism and arginine biosynthesis as the most enriched pathways. This is of significance as the role of arginine in the survival and adaptaion of *S. aureus* has been documented (Crow and Thomas, 1982; Liu et al., 1995; Zhu et al., 2009; Lindgren et al., 2014). Indeed, previous studies have shown the importance of arginine in the survival of *S. aureus* during infection as it utilizes it as a source of carbon and nitrogen, it also has been shown to have a pertinent role in biofilm formation as well as pH homestasis (Lindgren et al., 2014). Morever, recent report by Freiberg et al. have shown induction of antibiotic tolerance by *S. aureus* communities due to depletion of argninie (Freiberg et al., 2023). However, to the best of our knoweldge no direct report on the role of arginine in the antimicrobial resistance mechanism has been reported.

In conclusion, our findings demonstrate the utility of the dual functionality methanol method for concurrent extraction of proteins and metabolites from Gram positive bacteria. The findings also provide comparative data demonstrating differences in the proteomic and metabolomic profiling of MSSA and MRSA isolate. With the growing emphasis on the importance of conducting profling using a multi-omics approach to better understand the main drivers of resistance and identify novel targets for anti-microbials, the validation of single-phase extraction methods such as the one introduced shows great promises.

## Data availability statement

The data is deposited in the ProteomeXchange Consortium via the PRIDE (Proteomics Identifications Database), accession number PXD050411.

## Author contributions

SB: Conceptualization, Data curation, Formal Analysis, Methodology, Writing – original draft, Writing – review & editing. AG: Data curation, Formal Analysis, Methodology, Writing – review & editing, Writing – original draft. RN: Conceptualization, Funding acquisition, Methodology, Writing – review & editing. HA-H: Formal Analysis, Methodology, Writing – review & editing. LM: Methodology, Writing – review & editing. MH: Methodology, Writing – review & editing. NS: Conceptualization, Data curation, Formal Analysis, Methodology, Supervision, Writing – review & editing. AS: Conceptualization, Data curation, Funding acquisition, Methodology, Supervision, Writing – original draft, Writing – review & editing.
